# Supplementary material for: Effect of GO Additive in ZnO/rGO Nanocomposites with Enhanced Photosensitivity and Photocatalytic Activity
Source: Nanomaterials (Basel). 2019 Oct 11;9(10):1441. doi: 10.3390/nano9101441 (PMC6835891; doi:10.3390/nano9101441)
Supplement: Supplementary file 1 [file nanomaterials-09-01441-s001.pdf]

Supplementary Information

Article

Effect of GO Additive in ZnO/rGO Nanocomposites with Enhanced Photosensitivity and Photocatalytic Activity

Chatchai Rodwihok [1], Duangmanee Wongratanaphisan [2], Yen Linh Thi Ngo [1], Mahima Khandelwal [1], Seung Hyun Hur [1] and Jin Suk Chung [1,*]

[1] School of Chemical Engineering, University of Ulsan, Daehak-ro 93, Nam-gu, Ulsan 680-749, Korea; c.rodwihok@hotmail.com (C.R.); ngoyenlinh0912@gmail.com (Y.L.T.N.); mahimaiitr@gmail.com (M.K.); shhur@ulsan.ac.kr (S.H.H.)

[2] Department of Physics and Materials Science, Faculty of Science, Chiang Mai University, Chiang Mai 50200, Thailand; duangmanee.wong@cmu.ac.th (D.W.)

* Correspondence: jschung@ulsan.ac.kr

Content:

Table S1. Comparisons of photosensitivity and time-dependent photocurrent response between the present work and other reported UV detectors.

Table S2. Comparisons of photocatalytic activity between the present work and other reported research.

Figure S1. The thickness of prepared films by spray coating

Figure S2. Current of as-synthesized ZnO/rGO with bending radius; (a) ZnO, (b) ZnO/rGO (10%), (c) ZnO/rGO (20%), and (d) ZnO/rGO (30%).

Figure S3. Time-dependent absorption spectra of Methyl blue (MB) solution under visible light using (a) ZnO, (b) ZnO/rGO (10%), (c) ZnO/rGO (20%), and ZnO/rGO (30%) as a photocatalyst.

Figure S4. The photoluminescence spectra of pristine ZnO and as-synthesized ZnO/rGO

Table S1 Comparisons of photosensitivity and time-dependent photocurrent response between the present work and other reported UV detectors.

Structure	Substrate	λ_{UV} (nm)	UV intensity (W/cm^2)	Bias voltage (V)	Dark current (A)	Photosensitivity (I_{UV}/I_{DARK})	Response time (s)	Ref.
MgZnO/ZnO thin film	Glass	365	3.20×10^{-3}	4	~4.64×10^{-6}	~1.01	-	[1]
ZnO nanowires	SiO_2/Si	325	0.42×10^{-3}	1.5	~0.50×10^{-6}	<4	-	[2]
ZnO nanowires	SiO2/Si	300	2×10^{-3}	0.1	~12.70×10^{-6}	~1.51	0.2	[3]
	SiO_2/Si	500	19.50×10^{-3}	0.1	~12.50×10^{-6}	~1.40	0.3	[3]
ZnO nanostructures	p-Si	365	0.80	3	~3.50×10^{-6}	~1.71	-	[4]
Ti-doped ZnO thin film	glass	~365	2×10^{-3}	5	~15.00×10^{-9}	~6.80	135	[5]
ZnO/rGO nanostructures	glass	365	0.80×10^{-3}	2	~7.00×10^{-6}	4	44	[6]
ZnO/GO nanostructures	glass	368	0.80×10^{-3}	4	-	20.10	-	[7]
ZnO/rGO (20%)	transparent film	365	0.62×10^{-3}	2	3.98×10^{-9}	8.81	18.16	This work

Table S2 Comparisons of photocatalytic activity between the present work and other reported research.

Catalyst	Catalyst concentration ($g\ L^{-1}$)	Light source	MB concentration ($mg\ L^{-1}$)	Degradation rate (%) and time (min)	k_c (min^{-1})	Ref.
ZnO/GO (3%)	0.4	Metal halide lamp	10	~92% / 30	0.042	[8]
ZnO-g-C_3N_4/GO (50%)	0.3	Visible light	10	99% / 90	0.030	[9]
GO/ZnO (1:2)	0.4	UV light (254 nm)	5	94.5% / 60	-	[10]
ZnO/rGO (2.5%)	0.5	Mercury lamp (310-400 nm)	10	~80% / 120	0.012	[11]
ZnO NPs/rGO	0.3	Hg lamp (365 nm)	10	99.5%/ 180	-	[12]
ZnO/rGO	0.1	Mercury lamp (365-366 nm)	10	83%/ 10	-	[13]
ZnO/rGO	0.15	Hg lamp (365 nm)	5	88%/ 260	-	[14]
ZnO/rGO (1.5%)	0.2	Natural sunlight	5	82.3% / -	-	[15]
ZnO/g-C_3N_4 (500 ℃)	0.2	4 - Visible-light lamps (545 nm)	10	~99% / 180	~0.033	[16]
ZnO/rGO (20%)	0.2	Fluorescent lamp	10	93.78% / 60	0.0482	This work

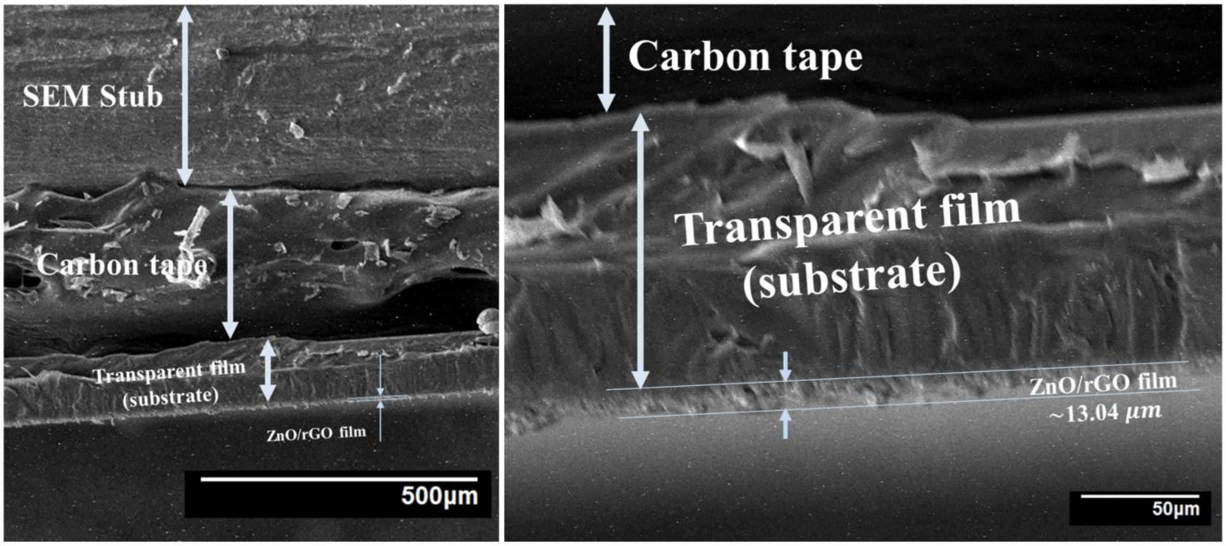

Figure S1. The thickness of prepared films by spray coating

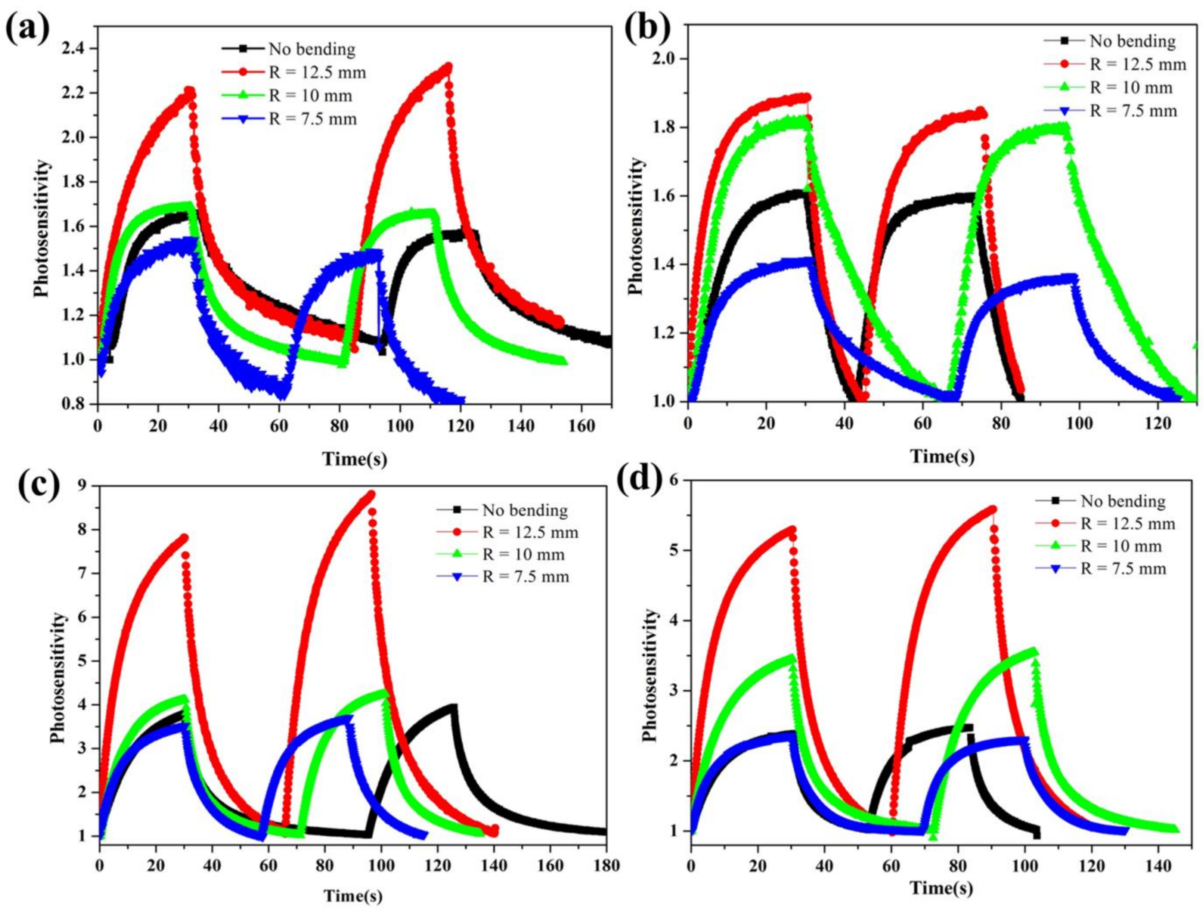

Figure S2. Current of as-synthesized ZnO/rGO with bending radius; **(a)** ZnO, **(b)** ZnO/rGO (10%), **(c)** ZnO/rGO (20%), and **(d)** ZnO/rGO (30%).

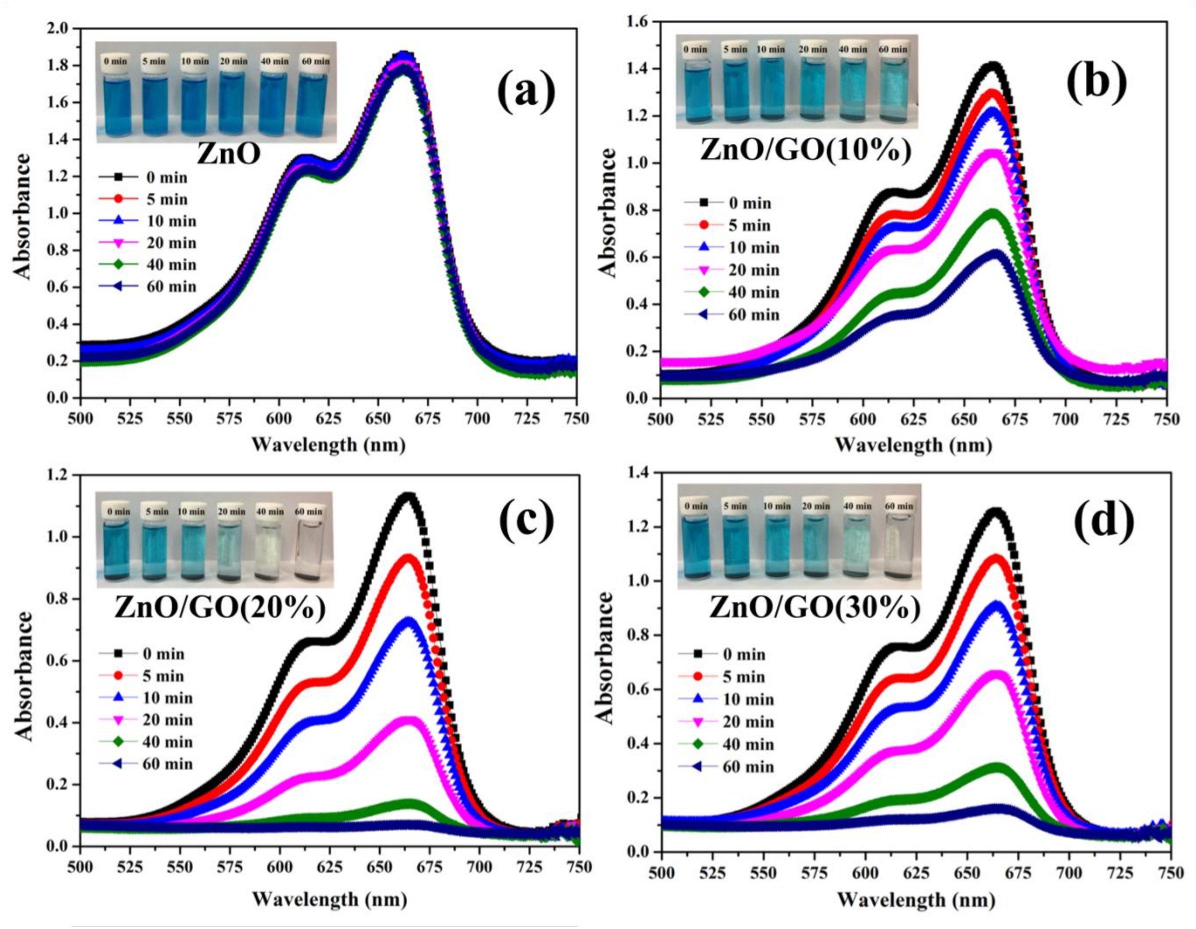

Figure S3. Time-dependent absorption spectra of Methyl blue (MB) solution under visible light using **(a)** ZnO, **(b)** ZnO/rGO (10%), **(c)** ZnO/rGO (20%), and **(d)** ZnO/rGO (30%) as a photocatalyst.

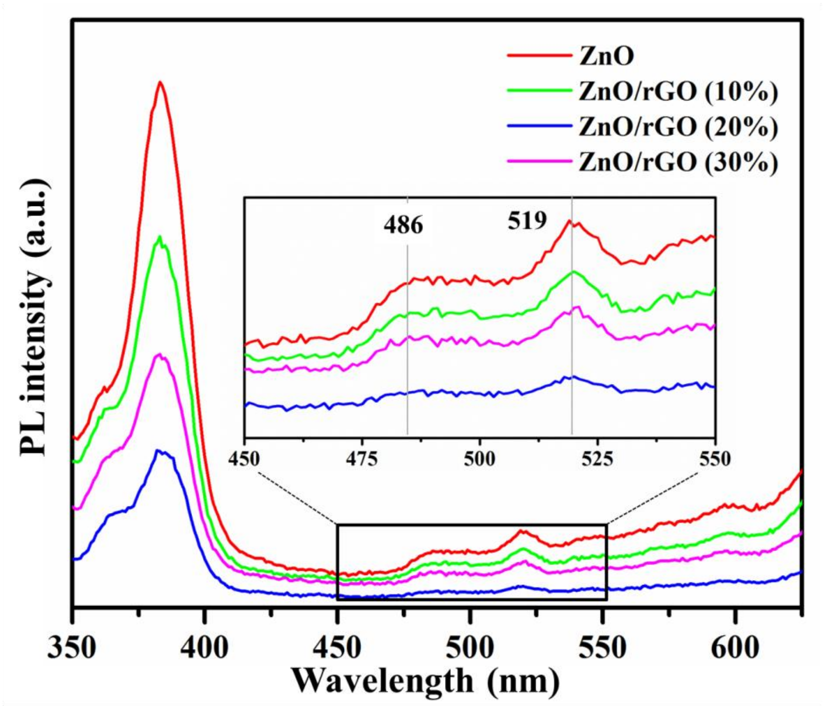

Figure S4. The photoluminescence spectra of pristine ZnO and as-synthesized ZnO/rGO

References

1. Rana, V. S.; Rajput, J. K.; Pathak, T. K.; Purohit, L. P., Multilayer MgZnO/ZnO thin films for UV photodetectors. *J. Alloys Compd.* **2018,** *764*, 724–729.
2. Lang, Y.; Gao, H.; Jiang, W.; Xu, L.; Hou, H., Photoresponse and decay mechanism of an individual ZnO nanowire UV sensor. *Sens. Actuators A Physical.* **2012,** *174*, 43–46.
3. Chao, L.-C.; Ye, C.-C.; Chen, Y.-P.; Yu, H.-Z., Facile fabrication of ZnO nanowire-based UV sensors by focused ion beam micromachining and thermal oxidation. *Appl. Surf. Sci.* **2013,** *282*, 384–389.
4. Bedia, A.; Bedia, F. Z.; Benyoucef, B.; Hamzaoui, S., Electrical Characteristics of Ultraviolet Photodetector based on ZnO Nanostructures. *phys. Procedia* **2014,** *55*, 53–60.
5. Shewale, P. S.; Lee, N. K.; Lee, S. H.; Kang, K. Y.; Yu, Y. S., Ti doped ZnO thin film based UV photodetector: Fabrication and characterization. *J. Alloy. Compd.* **2015,** *624*, 251–257.
6. Safa, S.; Sarraf-Mamoory, R.; Azimirad, R., Investigation of reduced graphene oxide effects on ultra-violet detection of ZnO thin film. *Phy. E: Low-dimens. Syst. Nanostructures* **2014,** *57*, 155–160.
7. Zare, M.; Safa, S.; Azimirad, R.; Mokhtari, S., Graphene oxide incorporated ZnO nanostructures as a powerful ultraviolet composite detector. *J. Mater. Sci Mater. in Electron.* **2017,** *28*, 6919–6927.
8. Qin, J.; Zhang, X.; Xue, Y.; Kittiwattanothai, N.; Kongsittikul, P.; Rodthongkum, N.; Limpanart, S.; Ma, M.; Liu, R., A facile synthesis of nanorods of ZnO/graphene oxide composites with enhanced photocatalytic activity. *Appl. Surf. Sci.* **2014,** *321*, 226–232.
9. Jo, W.-K.; Clament Sagaya Selvam, N., Enhanced visible light-driven photocatalytic performance of ZnO–g-C3N4 coupled with graphene oxide as a novel ternary nanocomposite. *J. Hazard. Mater.* **2015,** *299*, 462–470.
10. Munawaroh, H.; Sari, P. L.; Wahyuningsih, S.; Ramelan, A. H., The photocatalytic degradation of methylene blue using graphene oxide (GO)/ZnO nanodrums. In Proceeding of the AIP Conference Proceedings**, 2018,** 020119.

11. Jabeen, M.; Ishaq, M.; Song, W.; Xu, L.; Maqsood, I.; Deng, Q., UV-Assisted Photocatalytic Synthesis of ZnO-Reduced Graphene Oxide Nanocomposites with Enhanced Photocatalytic Performance in Degradation of Methylene Blue. *ECS J. Sol. State Sci. Technol.* **2017,** *6*, M36–M43.
12. Azarang, M.; Shuhaimi, A.; Yousefi, R.; Moradi Golsheikh, A.; Sookhakian, M., Synthesis and characterization of ZnO NPs/reduced graphene oxide nanocomposite prepared in gelatin medium as highly efficient photo-degradation of MB. *Ceram. Int.* **2014,** *40*, 10217-10221.
13. He, J.; Niu, C.; Yang, C.; Wang, J.; Su, X., Reduced graphene oxide anchored with zinc oxide nanoparticles with enhanced photocatalytic activity and gas sensing properties. *RSC Adv.* **2014,** *4*, 60253–60259.
14. Lv, T.; Pan, L.; Liu, X.; Lu, T.; Zhu, G.; Sun, Z., Enhanced photocatalytic degradation of methylene blue by ZnO-reduced graphene oxide composite synthesized via microwave-assisted reaction. *J. Alloys Compd.* **2011**, *41*, 10086–10091.
15. Omar, F. S.; Nay Ming, H.; Hafiz, S. M.; Ngee, L. H., Microwave Synthesis of Zinc Oxide/Reduced Graphene Oxide Hybrid for Adsorption-Photocatalysis Application. *Int. J. Photoenergy* **2014**.
16. Jung, H.; Pham, T.-T.; Shin, E. W., Interactions between ZnO nanoparticles and amorphous g-C3N4 nanosheets in thermal formation of g-C3N4/ZnO composite materials: The annealing temperature effect. *Appl. Surf. Sci.* **2018,** *458*, 369–381.
